# Acute intravascular hemolysis due to naphthalene toxicity: a case report 

**DOI:** 10.1186/s13256-018-1963-x

**Published:** 2019-03-22

**Authors:** A. A. A. Uthuman, C. S. Jayasinghe, A. H. N. Fernando

**Affiliations:** The National Hospital of Sri Lanka, Colombo, Sri Lanka

**Keywords:** Naphthalene, Mothball, Acute intravascular hemolysis

## Abstract

**Background:**

Naphthalene (mothball) is a commonly used deodorizer in the Indian subcontinent, including Sri Lanka. Though it is freely available around this country, poisoning has never been reported in the literature. Ingestion, either accidental or by deliberate self-harm, can occur due to its abundance as well as its candy-resembling appearance.

**Case Presentation:**

A 33-year-old Sri Lankan woman presented to us 2 days after the self-ingestion of 15 naphthalene balls. She had features of intravascular hemolysis without features of pigment nephropathy or methemoglobinemia. She was symptomatically managed with blood transfusion and adequate hydration.

**Conclusion:**

Naphthalene ingestion can lead to severe intravascular hemolysis as well as methemoglobinemia. The resultant pigment nephropathy may also lead to acute kidney injury.

## Introduction

Naphthalene, a simple polycyclic aromatic hydrocarbon, is commonly used as a deodorizer and moth repellent in the Indian subcontinent, including Sri Lanka. It is widely available in all grocery shops, street shops, and supermarkets. A mothball has the size of a jellybean, with its shiny and whitish appearance making it susceptible for accidental ingestion, mainly by children. The lethal dose of acute naphthalene toxicity is 5–15 g for adults and 2–3 g for children [1], with the standard weight of one mothball being 4 g. Overdose, either accidental or deliberate ingestion, can lead to a myriad of clinical manifestations, including hemolysis [2]. In Sri Lanka, there are no previously reported cases of naphthalene poisoning. Herein, we report on a patient with deliberate self-harm ingestion of 15 mothballs leading to severe hemolytic anemia warranting blood transfusion.

## Case Presentation

A 33-year-old married Sri Lankan woman presented with an episode of sudden onset of dark-colored urine with the background history of self-ingestion of 15 mothballs 2 days prior. This was an impulsive attempt after a quarrel with her husband. She denied co-ingestion of other substances including pharmaceuticals.

There was no significant complaint other than malaise and mild epigastric pain. She did not have features suggestive of urinary tract infection. Her past medical history, including history of hereditary hemolytic anemias, was unremarkable. She was not on any routine medications. Examination revealed severe pallor with lemon tinge icterus. Abdominal examination was normal, and other systemic examination was unremarkable.

**Fig. 1:**
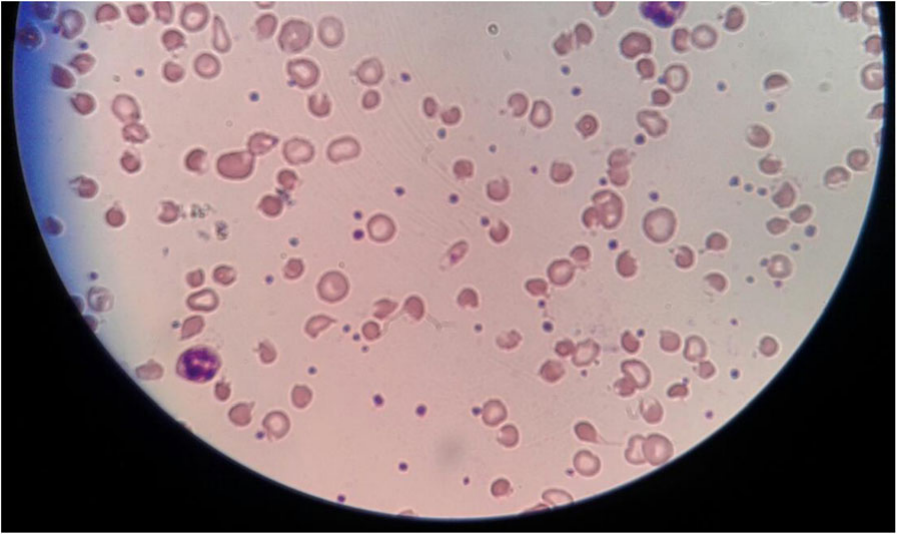
Acute intravascular hemolysis showing blister cells, bite cells, and red cell fragments

Her clinical test revealed severe normochromic normocytic anemia with a hemoglobin level of 5.9 g/dL and a reticulocyte index of 2.36 with indirect hyperbilirubinemia. Her blood picture featured normochromic normocytic red cells with reduced count, blister cells, bite cells, and red cell fragments suggestive of intravascular hemolysis (Fig. 1). Other investigations, including arterial blood gas are shown in Tables 1 and 2. As she had normal oxygen saturation and partial pressure, plasma methemoglobin levels were not measured.

**Table 1:** Sequential value of laboratory parameters during hospital stay

	Day 1	Day 2	Day 3	Day 6	Day 8
Hb (g/dL)	8.0	6.6	6.0	10.1	10.4
Total bilirubin (μmol/L)		250.5	88.9	27.2	24
Direct bilirubin (μmol/L)		10.7	9.5	4.6	7.6
Creatinine (μmol/L)		67	58	47	67

*Abbreviations: HB* Hemoglobin

**Table 2:** Arterial blood gas

pH	7.417
pCO_2_	32.5 mmHg
pO_2_	114.2 mmHg
SaO_2_	96.8%

*Abbreviations:* *pCO*_2_ Partial pressure of carbon dioxide, *pO*_2_ Partial pressure of oxygen, *SaO*_2_ Arterial oxygen saturation

She was hydrated adequately with monitoring of urinary output as well as serum creatinine. During hospital stay, she was transfused with two packs of red cell concentrate. Over a week, the hemoglobin levels increased and hemolysis settled. She never went into acute kidney injury. A review after 4 weeks revealed a hemoglobin level of 12.1 g/dL and she was symptom free.

## Discussion

Naphthalene (C_10_H_8_) is a volatile polycyclic hydrocarbon used as a deodorizer and moth repellent in households. Toxic effects had been reported through various modes of exposure, including inhalation, external skin contact, and ingestion [3]. Myriads of clinical manifestations have been reported, yet few have been tabulated [4] (Table 3).

**Table 3:** Systemic effects of naphthalene exposure

Gastrointestinal effects	
Nausea, vomiting, abdominal pain, diarrhea	
Renal effects	
Increased creatinine level, increased serum urea nitrogen level, hematuria, renal tubular acidosis	
Respiratory effects	
Congestion, Acute Respiratory Distress Syndrome (noted at 2 ppm)	
Neurologic effects	
Confusion, lethargy, vertigo, fasciculations, convulsions, anesthesia, cerebral edema, coma (coma is noted at 0.05 mg/kg body weight per day)	
Hepatic effects	
Jaundice, hepatomegaly, elevated liver enzyme levels (noted at 0.02 mg/kg per day)	
Ocular effects	
Optic atrophy, bilateral cataracts with chronic exposure	

Its toxic manifestations are mainly due to production of oxygen free radicals leading to lipid peroxidation and deoxyribonucleic acid (DNA) damage [5]. Hemolysis occurs usually in susceptible individuals such as in those who are G6PD deficient. In addition to hemolysis, due to its potent oxidizing property, it converts hemoglobin to methemoglobin, leading to methemoglobinemia [6]; therefore, the presence of cyanosis with normal oxygen saturation in arterial blood gas should raise the suspicion of methemoglobinemia.

Significant intravascular hemolysis gives rise to hemoglobinuria due to resultant saturation of hemoglobin scavengers such as haptoglobin. This may lead to acute kidney injury due to tubular precipitation of free hemoglobin [7], which was not seen in our patient. Hemolysis usually starts by the second day of exposure and can be protracted up until a week. Therefore, screening for hemolysis should continue until a week of post-exposure [8]; however, in this case, the patient presented with hemolysis features on the second day of ingestion.

A fall in hemoglobin and hematocrit levels with a high reticulocyte index, as well as spherocytosis and Heinz bodies in blood picture denote hemolysis. Unconjugated hyperbilirubinemia as well as high lactate dehydrogenase would also be found. Our patient’s smear revealed blister cells, bite cells, and red cell fragments (Fig. 1). Pigment nephropathy and acute kidney injury were prevented by adequate hydration and urine alkalization.

With regards to methemoglobinemia, the antidote would be methylene blue. However, an urgent G6PD enzyme assay is indispensable prior to the administration of methylene blue as it can paradoxically cause methemoglobinemia in G6PD-deficient patients [9].

## Conclusion

Naphthalene overdose can lead to severe intravascular hemolysis as well as methemoglobinemia. Both are potentially treatable when diagnosed promptly. Complications such as acute kidney injury could be prevented by meticulous fluid management and urinary alkalization. As it is extremely common in households, physicians should be aware of the toxidrome of naphthalene poisoning.
